# The Emergence of Quinolone Resistant *Shigella sonnei*, Pondicherry, India

**DOI:** 10.1371/journal.pone.0160290

**Published:** 2016-08-05

**Authors:** Ankita Das, Mailan Natarajan, Jharna Mandal

**Affiliations:** Department of Microbiology, Jawaharlal Institute of Postgraduate Medical Education and Research, Pondicherry, India; Baylor College of Medicine, UNITED STATES

## Abstract

Ciprofloxacin resistant *Shigella sonnei* across the globe have been increasing alarmingly. In order to understand the emergence of *S*.*sonnei* with respect to ciprofloxacin resistance in our patient population, the following study was carried out. Of the 184 *Shigella* sp. Isolated from 2012 to 2015, 34 *S*.*sonnei* which were confirmed by standard methods and subjected to antimicrobial susceptibility testing were selected. The minimum inhibitory concentrations (MICs) of 16/34 quinolone resistant isolates tested ranged from 4micrograms/ml to 16micrograms/ml for ciprofloxacin, from 16 micrograms/ml to 64 micrograms/ml for ofloxacin and from 16micrograms/ml to 64micrograms/ml for levofloxacin. Sequence determination of the quinolone resistance determining regions of *gyrA*, *gyrB*, *parC*, and *parE* genes showed mutations in GyrA at Gln69/Trp, Phe71/Ser, Ser72/Pro, Met75/Leu, Ser90/Cys, Met94/Leu, His106/Pro, Asn161/His, Thr163/Ala and in ParC at Ala64/Asp. Among the plasmid-mediated quinolone resistance (PMQRs) targets investigated,*qnrB* was the most (93.7%) prevalent followed by *qnrC* (18.7%). None had*qnrA*, *qnrS* and *qepA*. Two (0.1%) of the isolates harboured the*aac(6’)-lb* gene. Drug accumulation assay detected the presence of efflux pump activity in 9/15 (60%) among ciprofloxacin resistant isolates. All isolates harboured the *ipaH* gene followed by *ial* (17.6%), *sen* (11.7%), *set1A*&*set1B* (5.8%) genes. None had *stx1* element. PCR for Enterobacterial repetitive intergenic consensus (ERIC) sequences resulted in 4 unique clusters, of which Type III was the most (44%) dominant but there was no correlation between the ERIC types and the antibiotic resistance pattern or the virulence profile. A documented increase in *S*.*sonnei* harbouring the *qnr*genes and some unusual genes like *set1A*and indicate an ongoing process of horizontal gene transfer. The accumulation of novel mutations in GyrA and ParC in the presence of efflux pump and PMQR genes contributed to the raised MIC to quinolones. These findings are crucial in our understanding of quinolone resistance in these isolates.

## Introduction

Shigellosis is very contagious and can spread through communities at a fast rate posing a health threat globally. The low infective dose and the transmissibility of this agent through fingers, flies, fomites, food and faecal contamination of drinking water allows this organism to cause outbreaks very easily [1].

Shigellosis can be caused by any of the known serogroups of *Shigella*. But the relative frequency of the isolation of the most common serogroup differs geographically, the most common being *Shigella flexneri* in the developing nations. Though *S*.*sonnei* has been attributed to cause disease mostly in developed countries the current scenario speaks otherwise as it is marked by spread of this agent due to travel to endemic places. Several reports from the Indian subcontinent and south-east Asian countries have revealed a changing scenario regarding the shift in serogroups of *Shigella*. All in common have highlighted the growing importance of *S*.*sonnei* emerging as a close competitor of *S*.*flexneri* in its dominance [2,3,4]. Ciprofloxacin has been an empirical therapy for shigellosis across the globe for a very long time. Resistance to quinolones is mediated by mutations in the chromosomal genes called the quinolone resistance determining regions (QRDRs) of DNA gyrases namely, *gyrA* and *gyrB* and the Type IV topoisomerases namely, *parC* and *parE* or the presence of plasmid genes called the plasmid mediated quinolone resistance regions (PMQRs) namely the *qnr* (*qnrA*, *qnrB*, *qnrC*, *qnrD*, *qnrS*, *qep*, *aac(6’)lb-cr*) genes. Drug efflux pumps like *mdfA*,*tolC*,*ydhE*,*marA*. confer quinolone resistance. Any change in the outer membrane leading to porin loss can also result in increase in MIC to quinolones. The emergence of ciprofloxacin resistance has been reported since 2002 with the clonal spread of a ciprofloxacin resistant *S*.*dysenteriae* serotype 1 from Kolkata to other places followed by *S*.*flexneri* 2a and *S*.*flexneri* 3a in 2004 and finally in *S*.*sonnei* in 2007[2]. There are increasing reports regarding ciprofloxacin resistant *Shigella* across the globe which can be attributed to increased frequency in international travels, low infective dose and ease of accepting antibiotic resistance genes by horizontal gene transmission [5].

The following study was carried out to understand the emergence of *S*.*sonnei* with respect to ciprofloxacin resistance in our patient population.

## Materials and Method

The study was reviewed and approved by the Institute Ethics Committee (Human studies) at Jawaharlal Institute of Postgraduate Medical Education and Research (JIPMER), Pondicherry (Approval No. JIP/IEC/2014/5/324). Since, the samples are collected for routine diagnostics, our hospital does not have a policy for taking informed consent for collecting stool samples from patients. The ethics committee approval was obtained to store and study the microbial isolates obtained from these samples.

*S*.*sonnei* isolated from stool samples at Department of Microbiology, Jawaharlal Institute of Postgraduate Medical Education and Research (JIPMER), Pondicherry, India between years 2012 to 2015 were selected for this study. The isolates were obtained from stool samples of diarrhoea and dysentery cases and confirmed by standard biochemical tests [6] followed by serotyping by slide agglutination using specific antisera (Denka-Seiken, Tokyo, Japan). Antibacterial susceptibility and minimum inhibitory concentrations (MICs) of the isolates was determined in accordance with the Clinical laboratory standards institute (CLSI) guidelines[7] by the Kirby Bauer method and the MICs of quinolones was performed using the E-test(Biomerieux, Etoile, France). The quinolones tested included ciprofloxacin (Cf), ofloxacin (Of) and levofloxacin (Le). ATCC *Escherichia coli* ATCC 25922 was included as control in each test.

Total DNA was extracted from the isolates using the boiling lysis method[8]. Briefly, a single colony from overnight culture was suspended in 100microlitre sterile molecular grade water. This preparation was boiled at 100degC for 30minutes followed by flash cooling on ice for 5 minutes. The preparation was then centrifuged at 10000g for 5 minutes and supernatant transferred to a fresh tube and stored at -20degreesC.

Polymerase chain reaction (PCR) based detection of virulence genes (*ipaH*, *sen*, *set1A*, *set1B*, *ial*, *and stx1*) among the isolated *Shigella* spp. was performed using published primers [9,10,11] as described in Table 1. The QRDRs of *gyrA*, *gyrB*, *parC*, and *parE* genes were amplified using published primers (Table 1) as described earlier [12]. PCR was performed to amplify PMQR targets for the presence of four major groups of *qnr* determinants, *qnrA*, *qnrB*, *qnrC*, *qnrS* and two additional PMQR genes, *qepA* and *aac(6′)-Ib-cr*, using published primers [13] as described in Table 1. All primers used in this study were obtained from Bioserve India biotechnologies India private limited, India.

**Table 1:** Primers used in this study.

GENE		SEQUENCE (5’→3’)	REFERENCE
*gyrA*	FORWARD	TACACCGGTCAACATTGAGG	12
	REVERSE	TTAATGATTGCCGCCGTCGG	12
*gyrB*	FORWARD	TGAAATGACCCGCCGTAAAGG	12
	REVERSE	GCTGTGATAACGCAGTTTGTCCGGG	12
*parC*	FORWARD	GTCTGAACTGGGCCTGAATGC	12
	REVERSE	AGCAGCTCGGAATATTTCGACAA	12
*parE*	FORWARD	ATGCGTGCGGCTAAAAAAGTG	12
	REVERSE	TCGTCGCTGTCAGGATCGATAC	12
*qnrA*	FORWARD	ATTTCTCACGCCAGGATTTG	13
	REVERSE	GATCGGCAAAGGTTAGGTCA	13
*qnrB*	FORWARD	GATCGTGAAAGCCAGAAAGG	13
	REVERSE	ATGAGCAACGATGCCTGGTA	13
*qnrC*	FORWARD	GGGTTGTACATTTATTGAATCG	13
	REVERSE	CACCTACCCATTTATTTTCA	13
*qnrS*	FORWARD	GCAAGTTCATTGAACAGGGT	13
	REVERSE	TCTAAACCGTCGAGTTCGGCG	13
*aac(6′)-Ib-cr*	FORWARD	TTGCGATGCTCTATGAGTGGCTA	13
	REVERSE	CTCGAATGCCTGGCGTGTTT	13
*qepA*	FORWARD	AACTGCTTGAGCCCGTAGAT	13
	REVERSE	GTCTACGCCATGGACCTCAC	13
ERIC	FORWARD	ATGTAAGCTCCTGGGGATTCAC	15
	REVERSE	AAGTAAGTGACTGGGGTGAGCG	15
*ipaH*	FORWARD	TGGAAAAACTCAGTGCCTCT	9
	REVERSE	CCAGTCCGTAAATTCATTCT	9
*sen*	FORWARD	ATGTGCCTGCTATTATTTAT	11
	REVERSE	CATAATAATAAGCGGTCGC	11
*ial*	FORWARD	CTGGATGGTATGGTGAGG	11
	REVERSE	GGAGGCCAACAATTATTTCC	11
*stx1*	FORWARD	CTGGATTTAATGTCGCATAGTG	10
	REVERSE	AGAACGCCCACTGAGATCATC	10
*set1A*	FORWARD	TCACGCTACCATCAAAGA	11
	REVERSE	TATCCCCCTTTGGTGGTA	11
*set1B*	FORWARD	GTGAACCTGCTGCCGATATC	11
	REVERSE	ATTTGTGGATAAAAATGACG	11

PCR amplified products were then subjected to sequencing using an ABI 3700 sequencer (Applied Biosystem, Foster, CA, USA) at Bioserve India biotechnologies India private limited, India. Contig sequences generated were assembled and compared using the Basic Local Alignment Search Tool (BLAST) of the NCBI.

Drug accumulation assay was performed as described earlier [14]. Briefly, ciprofloxacin sensitive and resistant isolates of *Shigella* were grown in LB and harvested. The MIC of ciprofloxacin was determined on Mueller Hinton agar with and without Carbonyl cyanide m-chlorophenylhydrazone (CCCP) at a concentration of 20 mg/L using the Etest (Biomerieux, Etoile, France). A reduction in the MIC by 2–4 fold indicated the presence of an efflux pump activity.

ERIC PCR was performed according to Ranjbar et al [15] with some minor modifications viz. the gel electrophoresis was performed at 80 V for 1 hour instead of 4 hours. The ERIC-PCR profiles were normalized and analysed by PyElph (which is an open source Python based software for gel images analysis).

## Results

Of the 184 *Shigella* sp. isolated *S*.*flexneri* was the maximum (95/184 = 51.7%), followed by *S*.*sonnei*(34/184 = 20.6%),*S*.*dysenteriae*(7/184 = 3.7%),*S*.*boydii*(6/184 = 3.1%)and non-typable *Shigella*(42/184 = 22.2%). The year wise number of isolates are as mentioned in Table 2.

**Table 2:** Isolation of different serogroups during the period of study (2012–2015).

Year	*S*.*dysenteriae*	*S*.*flexneri*	*S*.*boydii*	*S*.*sonnei*	*NT*Shigella*	Total
2012	0	21(55.26)	1(2.63)	5(1.31)	11(28.94)	38
2013	3(7.31)	20(48.78)	1(2.43)	11(26.8)	6(14.63)	41
2014	1(1.88)	24(45.28)	1(1.88)	13(24.52)	14(26.41)	53
2015	3(5.76)	30(57.69)	3(5.76)	5(9.61)	11(21.15)	52
Total	7(3.80)	95(51.63)	6(3.26)	34(18.47)	42(22.82)	184

* NT: non-typable

Of the 34 isolates in our study, the invasive plasmid gene *ipaH* was found in all the isolates followed by the invasion associated locus gene *ial* in 17.6% of isolates and *sen* in (11.7%). The *Shigella* enterotoxin genes *set1A*&*set1B*were found in 5.8% of the isolates. No *stx1* element was found among the isolates studied.

Of the 34 isolates studied, 16 showed resistance to fluoroquinolones namely, ciprofloxacin, levofloxacin and ofloxacin. The MICs of 16/34 quinolone resistant isolates tested ranged from 4micrograms/ml to 16micrograms/ml for ciprofloxacin, from 16 micrograms/ml to 64 micrograms/ml for ofloxacin and from 16micrograms/ml to 64micrograms/ml for levofloxacin.

Sequence determination of the QRDRs of *gyrA*, *gyrB*, *parC*, and *parE*genes showed mutations in GyrA and in ParC. The various mutations detected are as shown in Table 3. Fisher’s Exact test and Chi square test did not correlate with the presence of mutation of amino acids and high MIC of 16micrograms/ml (p value = 0.518).

**Table 3:** Detailed description of the virulence profile, antibiotic resistance profile, MIC of the quinolones tested, efflux pump assay, PMQRs, mutation analysis of the QRDRs and ERIC types.

Sl.no	Isolate no	Virulence gene profile						Antibiotic resistance pattern	MIC values (micrograms/ml)			MIC values (micrograms/ml) of Cf with efflux pump inhibitor	MIC values (micrograms/ml) of Cf without efflux pump inhibitor	Antibiotic resistance gene profile						Mutation analysis				ERIC type by NJ
		*Shet1a*	*Shet1b*	*ial*	*sen*	*IpaH*	*Stx1*		Le	Of	Cf	Cf + CCCP	Cf—CCCP	*qnrA*	*qnrB*	*qnrC*	*qnrS*	*aac-6-cr*	*qepA*	*gyrA*	*gyrB*	*parC*	*parE*	
1	S1/12	-	-	-	-	+	-	A/NA/CO	0.75	0.19	0.38	0.38	0.38	-	-	-	-	-	-	-	-	-	-	III
2	S93/12	-	-	-	-	+	-	NA/CO	0.5	0.25	0.75	0.75	0.75	-	-	-	-	-	-	-	-	-	-	III
3	S95/12	-	-	-	-	+	-	NA/CO	0.75	0.75	0.25	0.25	0.25	-	-	-	-	-	-	-	-	-	-	III
4	S247/13	-	-	-	-	+	-	CF/CO	16	16	8	4	8	-	+	-	-	-	-	106 (H→P)	-	-	-	I
5	S390/13	-	-	+	-	+	-	A/CF/CO	32	32	16	8	16	-	+	-	-	-	-	72 (S→P) 75 (M→L) 94 (M→L) 106(H→P)	-	64 (A→D)	-	I
6	S391/13	-	-	+	+	+	-	A/CF/CO	32	32	16	4	16	-	+	-	-	-	-	69 (Q→W) 71 (F→S) 90 (S→C) 161(N→H) 163(T→A)	-	-	-	II
7	S415/13	-	-	-	-	+	-	A/CO(I)	0.38	0.5	0.75	0.75	0.75	-	-	-	-	-	-	-	-	-	-	IV
8	S715/13	+	+	+	+	+	-	A/CF/CO	16	16	4	6	4	-	-	+	-	-	-	72(S→P)	-	-	-	I
9	S750/13	-	-	-	-	+	-	CO	0.75	0.5	0.125	0.125	0.125	-	-	-	-	-	-	-	-	-	-	III
10	S839/13	+	+	+	-	+	-	A/CO	1	0.75	0.5	0.5	0.5	-	-	-	-	-	-	-	-	-	-	I
11	S774/14	-	-	+	-	+	-	CO	0.75	0.75	0.38	0.38	0.38	-	-	-	-	-	-	-	-	-	-	III
12	S604/14	-	-	-	-	+	-	CF/CO	64	64	8	4	8	-	+	-	-	-	-	-	-	-	-	IV
13	S353/13	-	-	-	+	+	-	CO	32	32	16	4	16	-	-	-	-	-	-	-	-	-	-	IV
14	S190/13	-	-	-	-	+	-	CO(I)	0.5	1	0.19	0.19	0.19	-	-	-	-	-	-	-	-	-	-	IV
15	S432/14	-	-	-	-	+	-	CF/CO	32	32	16	4	16	-	+	-	-	-	-	90 (S→C)	-	-	-	II
16	S587/12	-	-	-	-	+	-	CO	0.38	0.5	0.125	0.125	0.125	-	-	-	-	-	-	-	-	-	-	IV
17	S187/12	-	-	-	-	+	-	CF/NA/CO	16	16	4	4	4	-	+	-	-	-	-	-	-	-	-	IV
18	S359/13	-	-	-	-	+	-	CF	32	32	16	4	16	-	+	-	-	-		69(Q→W)	-	-	-	IV
19	S163/14	-	-	-	-	+	-	CO	0.094	0.094	0.38	0.38	0.38	-	-	-	-	-	-	-	-	-	-	II
20	S463/14	-	-	-	-	+	-	CO	0.38	0.38	0.75	0.75	0.75	-	-	-	-	-	-	-	-	-	-	IV
21	S523/14	-	-	-	-	+	-	CO	0.75	0.75	0.38	0.38	0.38	-	-	-	-	-	-	-	-	-	-	IV
22	S367/14	-	-	-	-	+	-	CF/CO	32	32	16	4	16	-	+	-	-	-	-	75(M→L)	-	-	-	II
23	S385/14	-	-	-	-	+	-	CO	0.38	0.38	0.25	0.25	0.25	-	-	-	-	-	-	-	-	-	-	II
24	S404/14	-	-	-	-	+	-	A/CF/CO	16	16	4	4	4	-	+	-	-	+	-	71(P→S)	-	-	-	II
25	S448/14	-	-	-	-	+	-	CF/CO	32	32	16	4	16	-	+	-	-	-	-	-	-	-	-	III
26	S798/14	-	-	-	-	+	-	Nil	0.38	0.5	0.75	0.75	0.75	-	-	-	-	-	-	-	-	-	-	III
27	S409/14	-	-	+	+	+	-	CF/CO	32	32	16	4	16	-	+	-	-	+	-	90 (S→C), 161(A→H)	-	-	-	III
28	S800/13	-	-	-	-	+	-	A/CO	0.38	0.38	0.75	0.75	0.75	-	-	-	-	-	-	-	-	-	-	III
29	S427/15	-	-	-	-	+	-	A/CF/CO	32	32	16	4	16	-	+	-	-	-	-	69 (Q→W)	-	-	-	III
30	S442/15	-	-	-	-	+	-	A/CF/CO	32	32	16	16	16	-	+	-	-	-	-	71 (F→S) 163 (T→A)	-	-	-	III
31	S511/15	-	-	-	-	+	-	CF/CO	32	32	16	4	16	-	+	+	-	-	-	75 (M→L) 161 (N→H)	-	-	-	III
	S548/15	-	-	-	-	+	-	CF/CO	32	32	16	16	16	-	+	+	-	-	-	72 (S→P) 161 (N→H) 163 (T→A)	-	-	-	III
33	S608/15	-	-	-	-	+	-	CO	0.125	0.125	0.25	0.25	0.25	-	-	-	-	-	-	-	-	-	-	III
34	S1068/14	-	-	-	-	+	-	A/CO	0.38	0.38	0.75	0.75	0.75	-	-	-	-	-	-	-	-	-	-	III
35	ATCC 25922	ND	ND	ND	ND	ND	ND	Nil	0.032	0.032	0.012	0.012	0.012	ND	ND	ND	ND	ND	ND	ND	ND	ND	ND	ND

ND = Not done; Le = Levofloxacin; Of = Ofloxacin; Cf = Ciprofloxacin; CCCP = Carbonyl cyanide m-chlorophenylhydrazone (CCCP); NJ = Neighbour joining; H = Histidine; P = Proline; S = Serine; M = Methionine; L = Leucine; Q = Glutamine; W = Tryptophan; F = Phenyalanine; C = Cysteine; N = Asparagine; T = Threonine; A = Alanine

**Fig 1:**
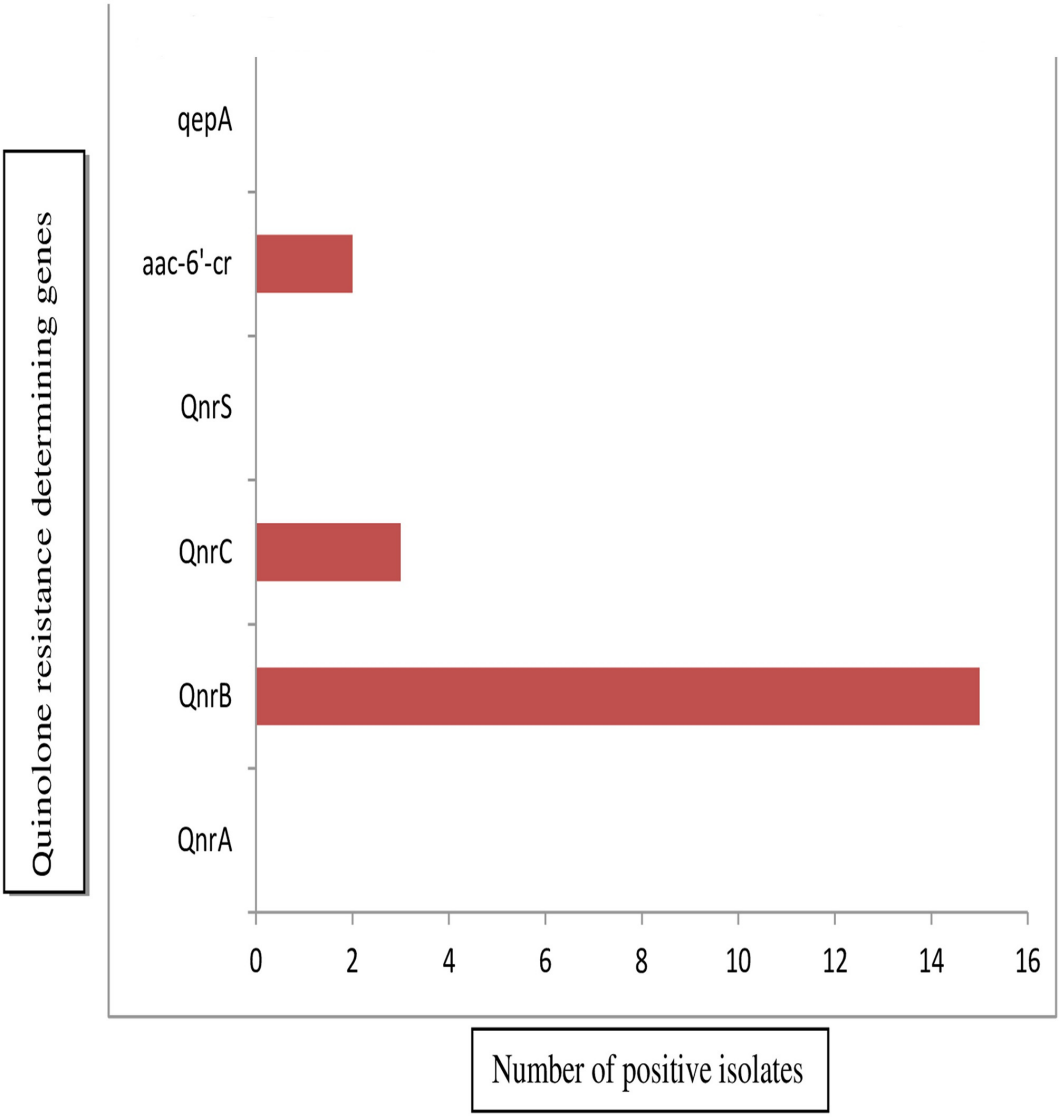
Distribution of Plasmid mediated quinolone resistance genes.

Among the PMQR genes,*qnrB* was the most prevalent, seen in 93.7% of the ciprofloxacin resistant isolates while, *qnrC* was positive in 18.7%. None harboured *qnrA* and *qnrS*. Two (0.1%) of the isolates were positive for *aac(6’)-lb-cr*gene. The *qepA* gene regulating the efflux pump was negative in all the isolates studied (Fig 1). Drug accumulation assay detected the presence of efflux pump activity in 9/15 (60%) among ciprofloxacin resistant isolates which showed fourfold decrease in MIC after the addition of CCCP. Spearman rank correlation coefficient and regression coefficient between PMQR gene and MIC of ciprofloxacin did not show any correlation.

None of the quinolone sensitive isolates harboured any of the PMQR genes. Though *gyrA*,*gyrB*, *parC*, *parE* was detected by PCR in them but none showed any mutation.

**Fig 2:**
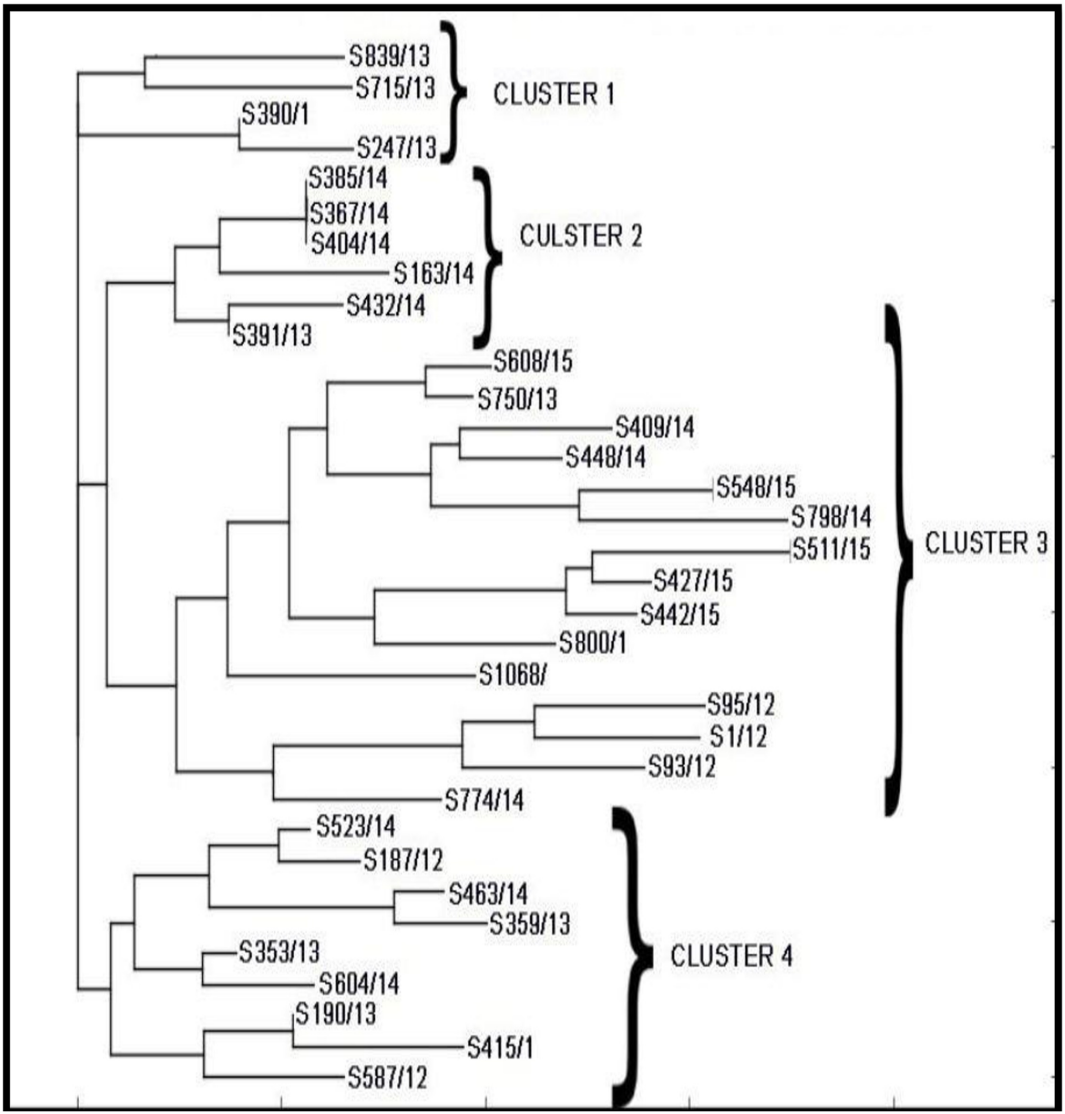
Phylogenetic tree using Neighbour joining method for ERIC pattern of *S*.*sonnei*.

All isolates were typable by ERIC-PCR. The ERIC PCR amplified DNA fragments ranged from 200 to 2500 bp. The number of bands produced ranged from 2 to 8, which were included in the statistical analysis because the bands were distinct in this region. The statistical analysis of the ERIC-PCR DNA fingerprints was performed with the PyElph graphic user interface program using hierarchical cluster analysis (Neighbour-joining method). ERIC–PCR analysis of the isolates resulted in four major ERIC groups (Table A in S1 File; Fig 2) labelled ERIC group I, II, III and IV. Type III was the dominant type (15/34 = 44.1%) followed by type IV(8/34 = 23.5%), type II and then type I(4/34 = 11.8%).

## Discussion

During the study period, *S*.*flexneri* remained the most dominant serogroup, as documented earlier [16,17]. The closest serogroup following this was the *S*.*sonnei* which showed a steep rise in 2013, maintaining a plateau till 2014 and then saw a downward trend (Table 2).

All the isolates had the *ipaH* gene with a variability in the presence of the other virulence genes namely, *ial*and *sen*. Overall, nearly 6% (3/34) of the isolates harboured both the *set1A* and *set1B* genes. The presence of *set 1A* is a very unusual finding in these isolates as this gene is not known to be present in this serogroup [18,19]. This enterotoxin gene is known to be present only in *S*.*flexneri*. Dissemination of this gene among the different serogroups needs to be studied in detail, though this unusual phenomenon could be attributed to horizontal gene transmission (HGT). Evidence for the presence of *set1A* and *set1B* genes in all *S*.*flexneri* in our sample population has been documented in an earlier published report [8]. HGT is well known in *Shigella* due to its genomic plasticity and the presence of mobile genetic elements which have been found very frequently in its megaplasmid. These 3 isolates showing the presence of *set1A* and *set1B* were isolated from 3 different patients, none of whom had any notable severity of infection.

Dysentery was noted in 24/34(70.58%) of the patients in the study group, while the remaining had complaints of watery diarrhoea. Of this 24, *ial* was detected in 2 (8.34%), *sen* in 3(12.5%), *set1A* and *set1B* in 1(4.16%) while *ipaH* was present in all. None of our isolates had *stx1*. A recent study from the USA reported *S*.*sonnei* producing Stx1 with clinical manifestations of bloody diarrhoea in the patients, suggesting a possible role of Stx1 with increased the risk of bloody diarrhoea in these patients [20]. Shiga toxin binds to the Gb3 receptors which are concentrated in the vascular endothelium, kidneys and brain but cannot act on the normal intestinal epithelium due to the absence of these receptors [21]. Contrarily, it can act on the malignant epithelium since it expresses Gb3 receptors[22].

In the present study we observed that 16(47.05%) were resistant to ciprofloxacin by the disc diffusion method and all these had high MIC of ciprofloxacin. All the 8 *S*.*sonnei* isolates from 2008 to 2011 were sensitive to ciprofloxacin, the difference being statistically significant(P value = 0.0159) (Table B in S1 file).

Meanwhile, we found that the MIC of all the other quinolones tested were also high in the isolates studied. Our findings are in concordance with those reported elsewhere [23,24] but unlike a recent study from Andamans, which had shown the MICs to be very high. This finding could be partly because of the presence of efflux pumps in some (27.27%) of these isolates [14]. The drug accumulation assay showed that majority (60%) possessed efflux pump activity, which may have additionally contributed to the increased MIC in our isolates. Further, an in-depth assessment in terms of detection and expression of efflux pumps like the *mdfA*, *tolC*, *ydhE*, *marA* etc., remains to be carried out.

We noted a few amino acid substitutions in the protein sequence of *gyrA* (Table 3). Mutations at multiple sites in *gyrA* lead to a higher increment in MIC of ciprofloxacin [25]. We did not find any mutation in *gyrB*. This has been documented earlier that majority of the mutations in clinical isolates have been detected in *gyrA* than *gyrB* [26]. We also detected mutation affecting a single amino acid in *parC*. It is noteworthy that the MIC is dependent on the specific amino acid substituted and the quinolone susceptibility is altered if a different amino acid is substituted at the same codon position [26]. Contrary to the usually reported mutations in GyrA Ser83 and Asp87 positions [27], we found novel mutations in the breakage reunion domain of GyrA which is known to be located between residues 67 to 106 (Table 3). Then we found two other mutations outside this domain which have not been reported elsewhere (Table 3). The most common mutation in ParC has been reported at Ser84 position but we found a mutation at position 64 (Table 3). These mutations accumulate in a step wise manner contributing to increase in MIC. Mutations were detected only in *gyrA* and *parC* but not in *gyrB* and *parE* in the ciprofloxacin resistant isolates. These are housekeeping genes and their mere presence do not signify anything and therefore it is important to look for mutations in these regions for quinolone resistance.

Among the PMQR genes, *qnrB* was the most prevalent in our study which is in concordance with earliest reports [28,29] while in contrast to results of other studies[30]. None of our isolates had *qepA*, which is a plasmid mediated efflux pump and *aac(6’)-lb-cr* gene which corresponds to aminoglycoside acetyl transferase. Qnr proteins by themselves contribute to the increase in MIC but also facilitate in selecting chromosomal mutants during therapy with a quinolone [28]. The MIC of ciprofloxacin did not correlate with the presence of the PMQR genes or the mutations in the QRDRs.

ERIC PCR has been found to be a practically useful alternative typing technique to PFGE for *Shigella* in small laboratories in developing countries. The major advantages are its simplicity, lesser turnaround time, cost per strain is less and need for specialized laboratory equipment is also less, besides, the results of ERIC PCR being comparable to that of PFGE [31,32]. To understand the degree of relatedness and look for any clustering among the isolates studied, ERIC PCR was performed in this study which resulted in 4 unique clusters, of which Type III was found to be of the most dominant with 44% of the isolates. The relative frequency of the ERIC type differed year wise. ERIC types III and II were significantly (p value 0.016) associated with high MIC of ciprofloxacin(16 micrograms/ml).

Foodborne associated outbreaks of *S*.*sonnei* were reported from Kerala and Maharashtra in 2009 and 2010, where clonal spread was observed with little difference in their pulse types [24]. In the year 2015, there were a few reports on outbreaks of ciprofloxacin-resistant *S*.*sonnei* from the USA and Korea [20, 23]. These outbreaks were due to travel into endemic areas where such infections are prevalent and also within day care centre contacts.

## Conclusions

Various mechanisms contribute to resistance, there being an occasional difference in the dominance of one over the other and it is not mandatory that all the mechanisms operate in a single strain at any given time. The horizontal transfer of plasmids carrying quinolone resistance determinants, the accumulation of chromosomal mutations and efflux pump played an important role in increasing rates of resistance in our isolates.

The study reflects the emergence of *S*.*sonnei* and quinolone resistance in them documented by the presence of novel mutations in target regions of the QRDRs. In addition, the presence of PMQR genes and efflux pump activity were also noted. The acquisition of an additional virulence gene found otherwise only in *S*.*flexneri* is an intriguing finding which may be reflective of a pathogen driven continuous evolutionary effort to adapt to the natural host environs. Hence alternative strategies aimed at disarming the bacteria by blocking their virulence rather than killing them or preventing their growth need to be developed.

## Supporting Information

S1 File **Table A,** Details of clusters of *Shigella sonnei* using ERIC PCR. **Table B,** Details of CIPROFLOXACIN resistant *Shigella sonnei* before and after 2011.
(DOCX)
