# Supplementary material for: The Emergence of Quinolone Resistant Shigella sonnei, Pondicherry, India
Source: PLoS One. 2016 Aug 5;11(8):e0160290. doi: 10.1371/journal.pone.0160290 (PMC4975386; doi:10.1371/journal.pone.0160290)
Supplement: S1 File — Table A, Details of clusters of Shigella sonnei using ERIC PCR. Table B, Details of CIPROFLOXACIN resistant Shigella sonnei before and after 2011. (DOCX) [file pone.0160290.s001.docx]

**Table A: Details of clusters of *Shigella sonnei* using ERIC PCR**

| **ISOLATE NUMBER** | **LANE NUMBER** | **ERIC TYPE BY N-J* METHOD** |
| --- | --- | --- |
| S1/12 | 1 | III |
| S93/12 | 2 | III |
| S95/12 | 3 | III |
| S187/12 | 17 | IV |
| S587/12 | 16 | IV |
| S190/13 | 14 | IV |
| S247/13 | 4 | I |
| S353/13 | 13 | IV |
| S359/13 | 18 | IV |
| S390/13 | 5 | I |
| S391/13 | 6 | II |
| S415/13 | 7 | IV |
| S715/13 | 8 | I |
| S750/13 | 9 | III |
| S800/13 | 28 | III |
| S839/13 | 10 | I |
| S163/14 | 19 | II |
| S367/14 | 22 | II |
| S385/14 | 23 | II |
| S404/14 | 24 | II |
| S409/14 | 27 | III |
| S432/14 | 15 | II |
| S448/14 | 25 | III |
| S463/14 | 20 | IV |
| S523/14 | 21 | IV |
| S604/14 | 12 | IV |
| S774/14 | 11 | III |
| S798/14 | 26 | III |
| S1068/14 | 34 | III |
| S427/15 | 29 | III |
| S442/15 | 30 | III |
| S511/15 | 31 | III |
| S548/15 | 32 | III |
| S608/15 | 33 | III |

***N-J : Neighbour joining**

**Table B : Details of CIPROFLOXACIN resistant *Shigella sonnei* before and after 2011**

|  | No of  *Shigella sonnei* ISOLATES FROM 2008-2011 | No of  *Shigella sonnei* ISOLATES FROM  2012-2015 |
| --- | --- | --- |
| TOTAL | 8 | 34 |
| CIPROFLOXACIN RESISTANT | 0 | 16 |
| CIPROFLOXACIN SENSITIVE | 0 | 18 |
